# PLEIAData: consumption, HVAC, temperature, weather and motion sensor data for smart buildings applications

**DOI:** 10.1038/s41597-023-02023-3

**Published:** 2023-03-03

**Authors:** Antonio Martínez Ibarra, Aurora González-Vidal, Antonio Skarmeta

**Affiliations:** Department of Information and Communication Engineering, University of Murcia, Murcia, 30100 Spain

**Keywords:** Energy efficiency, Energy management, Electrical and electronic engineering

## Abstract

The current cost that energy represents is crucial in a field like climate control which has high energy demands, therefore its reduction must be prioritized. The expansion of ICT and IoT come with an extensive deployment of sensors and computation infrastructure creating an opportunity to analyze and optimize energy management. Data on building internal and external conditions is essential for developing efficient control strategies in order to minimize energy consumption while maintaining users’ comfort inside. We here present a dataset that provides key features that could be useful for a wide range of applications in the context of modeling temperature and consumption via Artificial Intelligence algorithms. The data gathering has taken place for almost 1 year in the Pleiades building of the University of Murcia, which is a pilot building of the European project PHOENIX aiming to improve building energy efficiency.

## Background & Summary

The last decades, energy consumption and emissions derived from it have been a problem that needs to be addressed with new methods for energy efficiency. Among the sectors in which energy consumption is broken down, those being industry, transport and buildings, the latter account for approximately 40% of it^1^. Buildings, of any kind, have needs such as lighting, Heating Ventilation Air Conditioning (HVAC), electrical appliances, etc. HVAC systems are responsible of 50% of building expense and 15–20% of the total consumption^2^ and since they are easy to act upon them, control strategies can be developed in order to reduce energy expense^3^. The expansion of the IoT paradigm provides a scenario where buildings are equipped with sensors and smart meters allowing the collection of useful data and information^4^. With this data, it is possible to design Machine Learning models to get a better understanding of a myriad of phenomenons.

The steps carried out to deal with the datasets are the following:

- Data cleansing: problematic samples are removed and/or replaced.
- Descriptive analysis: understanding the data is crucial so we perform a descriptive analysis to visualize relationships among features, looking closely to the correlation matrix.
- Datasets union: since we work with several datasets, once they have been processed they are merged into only one dataset with some additional steps, like adding variables and setting an index to have samples in uniform intervals.

In this line of work, where the focus is energy consumption and the development of control strategies, there are lots of available public datasets. For example, in^5^ a transfer learning approach is taken, using data from buildings in different regions via various open datasets. First, from The Building non-residential Data Genome Project^6^ where data is collected in 1238 buildings mainly from the United States, but also from Europe, Asia and Oceania. This data includes physical features such as the number of floors and their area, hourly consumption in those buildings and meteorological variables like temperature and humidity, in addition to the primary use of the building, which mostly are universities and schools. Similarly, in^7^ minute to minute energy consumption is monitored as well as occupation in 10 minutes interval and weather variables, this time in 52 buildings in Asia. Lastly, in^8^ behavioural data related to some office workers is collected over a year.

Another example can be seen in^9^ where consumption related to more aspects than air conditioned is treated, adding data from lighting and plug loads, but this time only from one building. The others features from the dataset are indoor data: temperature, relative humidity and ambient light. In this article special attention is paid to the devices used for the measurements, to evaluate the quality of the data and also to make a descriptive analysis.

What this datasets provides, other than energy consumption, indoor temperature and weather, is HVAC information, highlighting the setpoint established by the users which we have not found in other studies. However, several research works have shown interest in this kind of data in order to improve demand response in smart buildings^10,11^. Apart from that, CO_2_ and presence data gathered via a motion sensor is also presented. On the other hand, the data is only from one building and the presence which could be useful for indoor temperature forecasts is only available for a small subset of all the rooms in the building.

The dataset can be used is many cases, such as:

- Forecasting the hourly building energy consumption time series via machine learning algorithms^12^.
- Selecting a subset of the features that are most valuable to develop energy forecasts and evaluate their quality^13,14^.
- Sending personalised recommendations to HVAC users use with the goal of reducing energy consumption^15^.
- Forecasting indoor temperature per room^16^.
- Developing a demand flexibility strategy coupling consumption and temperature forecasts^17^.
- Applying transfer learning to not sensorized or partially sensorized buildings with similar characteristics^5^.
- Implementing reinforcement learning techniques adding, for example, electricity prices to this dataset^18^.

## Methods

**Fig. 1:**
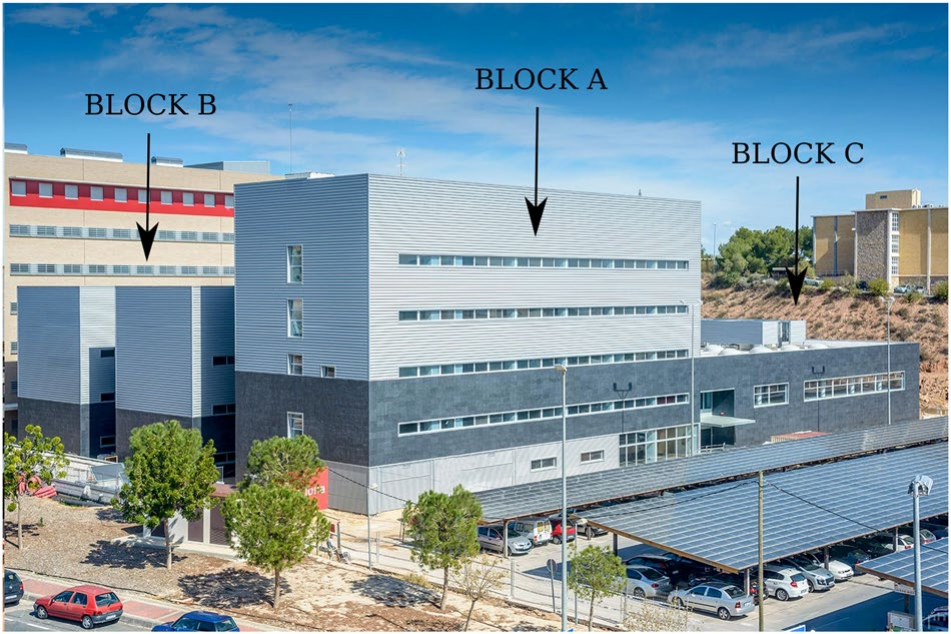
Pleiades, University of Murcia^20^.

The data collection takes place on the Pleidades building of the University of Murcia, a building composed of three blocks, shown in Fig. 1. This building is a pilot of the project *Adapt-&-Play Enhanced Cost-Effectiveness and user-friendliness Innovations with high replicability to upgrade smartness of existing buildings with legacy equipment* (PHOENIX), funded by the European Union’s Horizon 2020 Programme under Grant Agreement 893079. Pleiades consists of three buildings: the first building has five floors, the second has two and the third has only one, in addition to a ground floor for each, with a total area of 10,983 m^2^. The monitored areas include offices, laboratories, lecture halls, and libraries. According to the blueprints the area of the rooms ranges from 12 to 30 m^2^ and each floor has a height of 3.5 m^19^.

In this case scenario, integration with the different types of external data sources is considered^20^:

- PHOENIX External Data Source Adapter to retrieve weather data from external data sources. A middleware agent is being developed to enable the access to the APIs of the data source and send this information to the PHOENIX hub using a MQTT/SSL/ NGSI-LD interface. This agent runs on docker and its software requirements are 1 CPU core, 1 GB of RAM and 100MB of HD space.
- PHOENIX Building System Adapter to integrate with the BMS in UMU premises which is based on IoT platform “ Pleiades Building”. Two software modules have been deployed to track data from the BMS system. This adapter is composed of several docker containers which require 2 CPU cores, 2GB of RAM and 100MB of free HD space.
- PHOENIX IoT Adapter to integrate with 3rd party sensors or other systems available in several zones of the UMU site. In this case, the PHOENIX IoT Adapter is running in the local/demo environment through the deployment of several hardware components that are able to run this software implementation. The pilots are going to use a Raspberry Pi device (Raspberry Pi 3B+) as the IoT Gateway along with the USB Z-Stick Gen5 to ensure Z-Wave Integration of sensors and actuators. In case a MODBUS device is available, a software agent is also deployed at the local hardware (along with the appropriate hardware component in case of RS-485 connection) to track communication via MODBUS.

The hardware equipment for the domains consists of HVAC systems with actuation support at a split level whose functioning parameters (mode, set-point, etc) can be changed by each room user using a remote control, meters for electrical consumption at building level (kWh) since the HVAC system is centralized by building, and indoor characteristics like temperature (°C). Then, the devices are connected in the PHOENIX Smartness Hub^21^. The equipment for monitoring and control consist of three phase & one phase smart meters Zwave (Unit Schneider PM710), those being Qubino 3-Phase Smart Meter, Aeotec Home Energy Meter - three clamps GEN5, Zipato Energy Meter – 1 phase (2 circuits) and WiDom Energy Driven Switch C version. Temperature and humidity monitoring devices of the heating and cooling system are ZWave sensors - MCOHome MH9 and the HVAC units are Toshiba VRF, handled by smart control devices Intesis Box WMP – Universal IR. Lastly, the Gateway for monitoring and control is a Raspberry Pi 3 Model B + with Z-Wave Aeotec Z-Stick Gen5 USB.

In the Pleiades building there is a Building Management System (BMS) that is managing a number of devices. It has direct control over individual air conditioning units, temperature, humidity, lighting, etc and the data produced comes in JSON format^20^. The amount of data produced within the project is very high, because in the buildings there are hundreds of devices sending sensor data every 10 minutes approximately^22^.

**Fig. 2:**
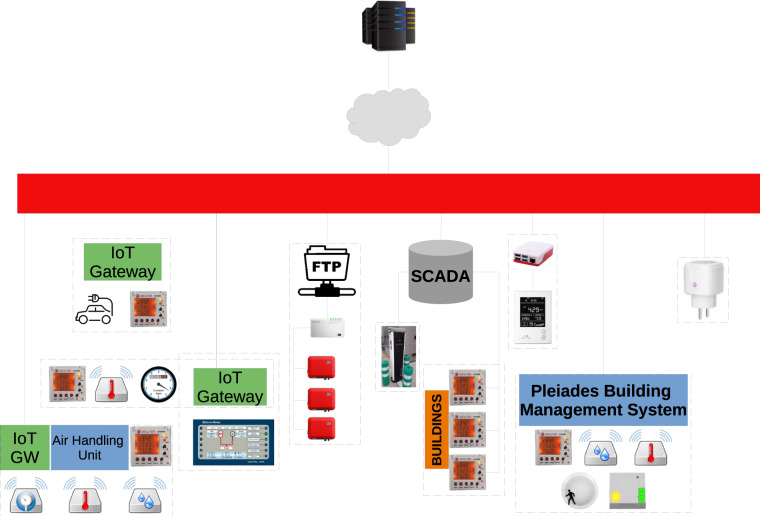
Architecture diagram UMU Pilot^26^.

Two major changes have been made in the integration of the Pleiades BMS of the UMU Pilot with respect to other PHOENIX pilots. First in actuation support, which has been implemented using the Context Provider concept offered by the Context Broker. Then, improvements to reduce the lag detected in certain situations. Once the actuation support was added, there are certain situations in which the delay between sending an actuation and getting the status of the devices updated was not acceptable. The most obvious case is the air conditioning system, where too big delays might interfere the normal operation of certain services related to air quality or comfort, for example. These delays can be even bigger as the 5 splits from the pilot are inside a bigger group that is controlled by a single gateway integrated in the BMS, and the access to all the splits of the building is cyclic, so reading or writing values to one of them requires the control process of the gateway to reach this specific one, which might take some time. Knowing how the BMS works with this type of devices, the connector has been updated to work with the splits asynchronously in order to send the read/write requests faster, but only for them as it is still working synchronously with all the other devices Fig. 2.

**Fig. 3:**
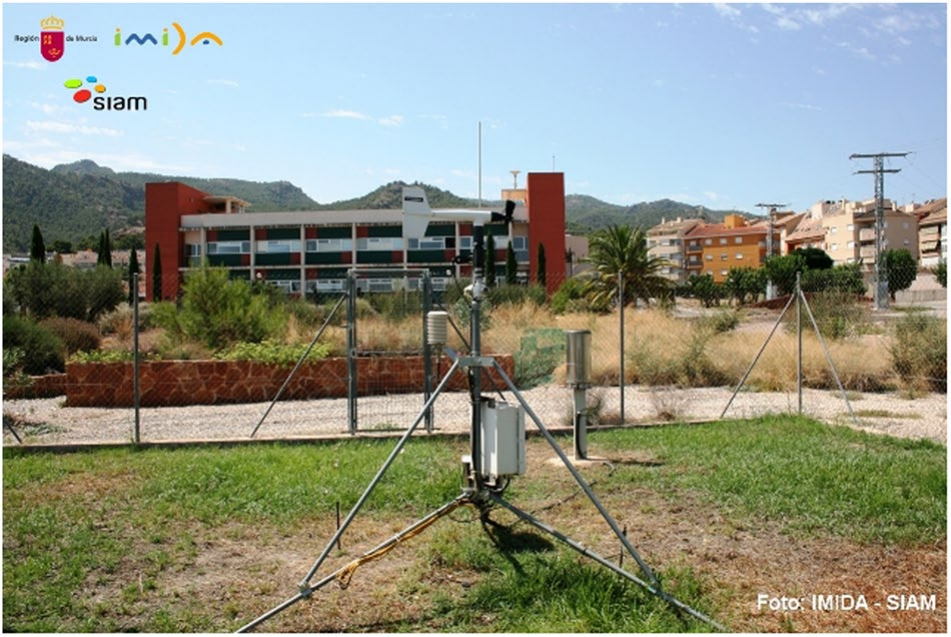
MU62 station^23^.

The external data, that is, weather features come from the *Instituto Murciano de Investigación y Desarrollo Agrario y Medioambiental* (IMIDA^23^) and *Sistema de Información Agrometeorológica de la Región de Murcia* (SIAM^24^), a transfer organization between the scientific community and the productive sector. This organization provides an network of agrometeorological stations to gather and transfer information. In Fig. 3 the MU62 station of which we used the data is shown:

## Data Records

The entire dataset is divided in 9 *csv* files and 1 *txt* file with data gathered from January 1, 2021 to December 18, 2021 and it is available in a Zenodo repository^25^ in the *raw_data* folder. Taking into account that all those datasets are in tabular form, meaning that each row of the *csv* is an observation and each column is a feature, those files can be easily imported in any programming language and are listed in Table 1.

**Table 1:** Listed files and size.

File	Size (MB)
*data-cons.csv*	12.3
*data-sensor.csv*	492.9
*data-hvac.csv*	257.7
*data-CO2.csv*	548.8
*data-presence.csv*	12.2
*MU62_dm.txt*	6.5
*relations-sensor.csv*	0.006
*relations-hvac.csv*	0.007
*relations-CO2.csv*	0.001
*relations-presence.csv*	0.001

First, the consumption dataset (*data-cons.csv*) has 154833 rows and 4 columns with samples for 3 block identifiers every 10 minutes approximately (sampling time is not exactly uniform). Consumption data has four features:

- ID: unique identifier for each observation.
- IDdevice: identifier with 3 possible values, each referencing a block of the building (A, B or C): 335546928 (A), 335546926 (B), 335546927 (C).
- Date: timestamp with format %Y-%m-%d %H:%M:%S.
- V22: aggregated consumption by block of all the monitored HVACs, since there is no specific smart meter associated to each room.

Second, the indoor temperature dataset (*data-sensor.csv*) has almost 10 M rows (9857796) because it has a value every 10 minutes for 191 sensors during a 1 year period. This dataset only contains 3 columns:

- IDdevice: identifier of the sensor and related to a room by the relationships explained a few lines below.
- Date: timestamp with format %Y-%m-%d %H:%M:%S.
- V2: temperature in °C.

Then, HVAC monitor (*data-hvac.csv*) deals information about several aspects of the device operation:

- IDdevice: identifier of the HVAC device.
- Date: timestamp with format %Y-%m-%d %H:%M:%S.
- V4: state of the HVAC which can take the value 0 or 1 if the system is off or on, respectively.
- V5: references the operation mode of the device between the values 0, 1 and 2 representing none (if the device is off), heat or cold.
- V12: setpoint temperature established by the user.
- V26: type of device, which can be wall (1) or ceiling (2).

Like the temperature dataset, this file is also large, with 4339076 entries, again one sample approximately every 10 minutes for 88 HVAC devices during the same year.

Next, weather data (*MU62_dm.txt*) has only 55 K rows with the following features:

- Fecha: date with format %d%m%y.
- hour woth format %H%M%S.
- tmed, tmax, tmin: mean, max and mininmum values of temperature in °C.
- hrmed, hrmax, hrmin: relative huminity in %.
- radmed, radmax: solar radiation in W/m.
- vvmed, vvnax: wind speed in m/s.
- dvmed: wind direction measured in grades from the north.
- pred: precipitation in l/m.
- dewpt: dew point in °C.
- dpv: vapor pressure deficit in kPa.

It should be noted that this datasets presents a slightly different date format due to it coming from a source outside the university but the measurements are also taken every 10 minutes. This will be addressed in the following section.

It is crucial being able to tell which temperature sensor and HVAC device is located in which room and therefore, their relationships are gathered in *relation-sensors.csv* and *relations-hvac.csv*. These datasets present the same structure, both with 4 columns and 90 and 86 rows, respectively. Those columns are:

- ID: identifier of the HVAC/sensor device.
- desc: text description of the device’s location.
- block: string with the block in which the device is, A, B or C.
- room: number of the room in which the device is.

As we can see, the number of rooms in the relationships and the number of sensor and HVAC devices is not the same in all datasets and the number of sensor and HVACs do not match. This is because not all rooms have both sensor and HVAC, and some rooms have two of those instances. This must be taken into consideration when the data is treated in a room level granularity.

Carbon dioxide measurements are available only for a few rooms, and in this dataset we have the identifier, the date and the CO_2_ value, in parts per million (ppm). This is a large dataset because the sampling rate is approximately 10 minutes and it has registers of other types of devices, not only CO_2_. Likewise, for presence we have the identifier, data and presence as boolean. Presence is labeled as V2 by default, which was already the label for temperature in its respective dataset, so when the data is treated one of those two should be relabeled to avoid confusion.

And just like we had relationships for temperature and HVAC, there are also relationships for the CO_2_ and presence monitoring devices. In *relations-CO2* we have the identifier, room and block of each one of the CO_2_ measuring appliances and in *relations-presence* room and block, this time from the motion sensors, along with a brief description of the room.

## Technical validation

The following subsections show a descriptive analysis of the datasets and how they can be treated so they are ready to be used in the development of a machine learning model or any other application. All the steps below are accessible via the notebook “Notebook_Scientific_Data_Nature.ipynb” that can be downloaded at^25^. This repository also includes the aforementioned folder *raw_data* where the reader can find the raw data and the processed data that is the output of the following sections in the folder *processed_data*.

### Consumption dataset

**Fig. 4:**
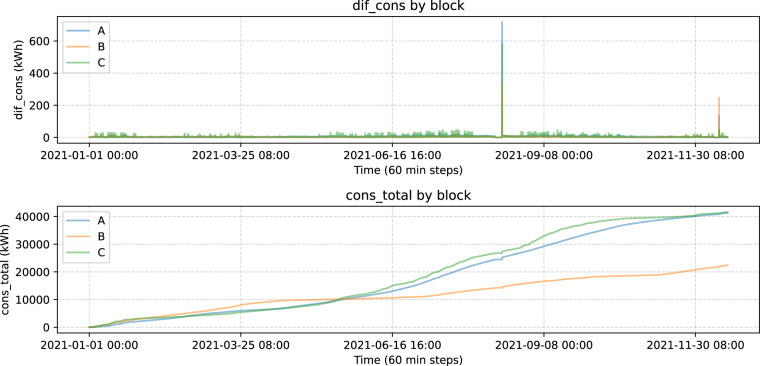
Punctual and total consumption.

**Fig. 5:**
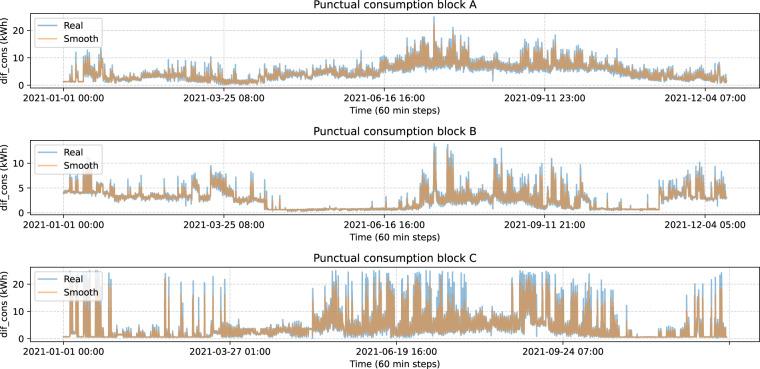
Real and smoothed consumption by block.

**Fig. 6:**
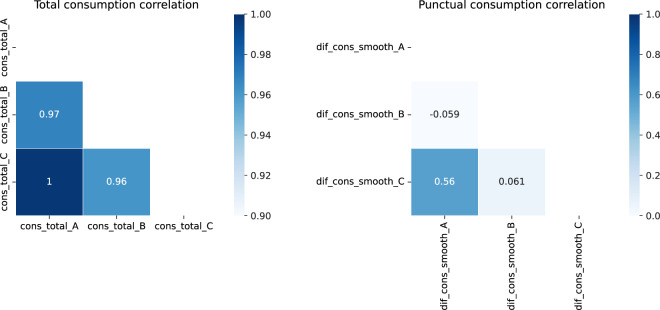
Total and punctual correlation.

The first step with this dataset consists of separating the samples by building block and resample so we have uniform time steps of 60 minutes. This is the interval that we chose to portray the characteristics of our dataset because it leads to an easier understanding and also due to 10 minutes intervals being too close to reflect significant consumption changes and are more affected by noise. Apart from this, we added a column accounting for the total consumption from the start to the end of the data gathering and another column that is the consumption difference between each time step and the previous, denoted as *dif_cons* and we will refer to it as punctual consumption. Punctual consumption and total consumption for blocks A, B and C are shown simultaneously in Fig. 4:

Here we find two irregularities that affect the three blocks. These two events are a set of 0’s lasting for over 300 observations followed by a huge peak. In the lower plot, there is a flat interval between 21-06-16 and 21-09-08 (2021-08-12 to 2021-08-15) and the same phenomena can be observed (although it is not that noticeable) after 2021-11-30 (2021-12-10 to 2021-12-11). Then, after those intervals in the upper plot we see the two mentioned peaks. Given that the reason for this phenomenon is unknown to us, we decided to remove observations larger than 25 or equal to 0 for the descriptive analysis, however, the raw data is provided so that researchers and practitioners can make their own decisions with regard to this. Smoothing or interpolation is not useful in this situation because with so many zeros that interval ends up being constant which is not what we expect from a consumption standpoint. Once we do that, there are no extreme peaks like before, but it seems like there is a lot of noise. To solve that we do a smoothing with *dif_cons*. Now, in Fig. 5 we see real and smoothed consumption without extreme peaks for the three blocks, plotting each block individually:

Lastly, we plot in Fig. 6 correlations for accumulated and punctual consumption between blocks.

While total consumption is highly correlated, that is not the case with punctual consumption. Therefore, the implementation of this dataset into a forecast model for consumption may should be split in three models, one for each block.

### Temperature dataset

**Fig. 7:**
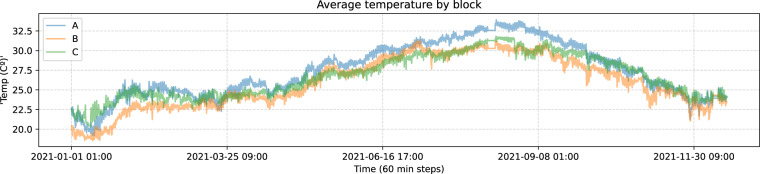
Average temperature by block.

**Fig. 8:**
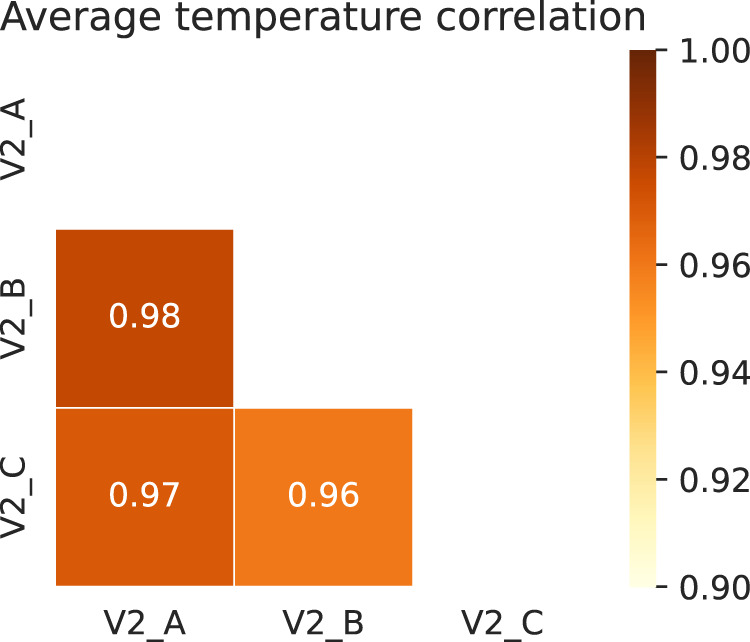
Average temperature correlations.

As before, with this dataset we will also perform a resample in 60 minutes intervals taking the mean temperature. Now, if we take a look at the distribution of the dataset we will come across several errors, with extreme values that do not make sense, for example, temperatures lower than 0 °C or greater than 60 °C inside the rooms. This values are replaced by the last valid value. Once we have done that, we represent average temperatures by block in Fig. 7 and their correlation in Fig. 8:

As we observe in both plots, average temperatures behave similarly in the three blocks, whereas the consumption behaves quite differently. Based on that, implementing this into a temperature forecast would allow us to develop a model for the whole building using all the data at once.

### HVAC dataset

**Fig. 9:**
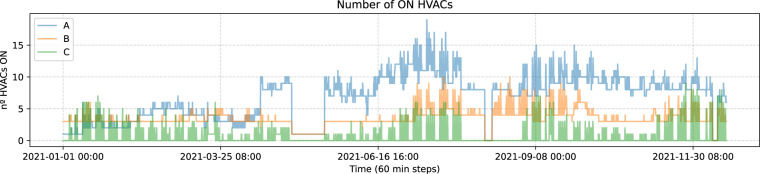
Number of on HVACs by block.

The HVAC dataset, having more features than the other requires more work to prepare. The steps that we will follow this time are: (i) Replace extreme values of V12, like we did with V2 in temperature; (ii) Resample V4 to get the number of turned on HVACs; (iii) Resample V12 to get the mean setpoint temperature; (iv) Resample V5 to get the predominant operation mode; (v) Resample V26 to get a mean with the device type; (vi) Merge those resamples; (vii) Add a feature to split the day in three parts (morning/afternoon/night); (viii) Add a feature to split the year in seasons. Then, we plot the number of turned on HVACs during the monitor period by block in Fig. 9.

Looking at this, we see a behaviour that makes sense, since in summer (around observation 4000) and then closer to the winter more HVACs are being used, while in spring, when temperatures are more comfortable and there is no need to turn on cooling or heating devices there are not that many on instances.

### Weather dataset

**Fig. 10:**
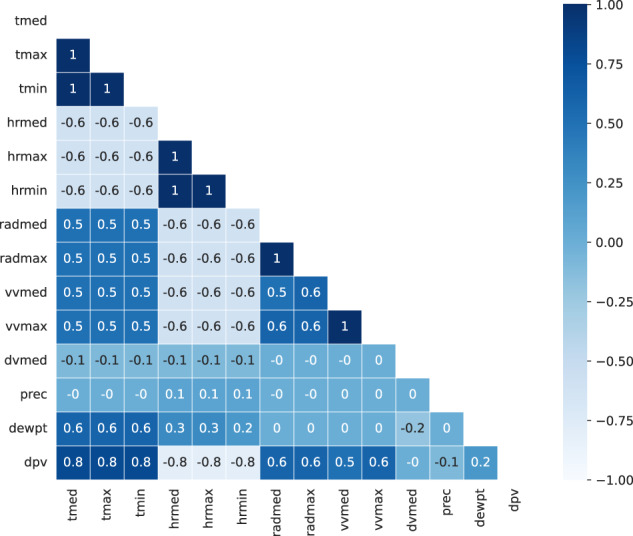
Weather correlations.

The weather dataset has a couple of features that intuitively should be highly correlated, and therefore, do not bring much information when it comes to train a model. Those being several temperature, humidity and radiation measurements. We have decided to plot in Fig. 10 the correlations between the variables of this dataset to show the correlation between them so that it would ease the dataset user choice when deciding what variables to use or explore:

We see that there are groups of variables totally correlated. As a consequence, we select one of each group, so from *tmax, tmin* and *tmed* we pick *tmed*, from *hrmax*, *hrmin* and *hrmed* we pick *hrmed*, from *radmed* and *radmax* we pick *radmed* and from *vvmed* and *vvmax* we select *vvmed*. Next, another resample is performed taking the mean value and we also change the date (*fecha* & *hora* fields) format so it is the same as the other datasets (%Y-%m-%d %H:%M:%S).

At this point we have every dataset ready but separated. Here we propose two types of merge, one with aggregates by block and one at room level granularity.

### Dataset merge 1. Consumption

**Fig. 11:**
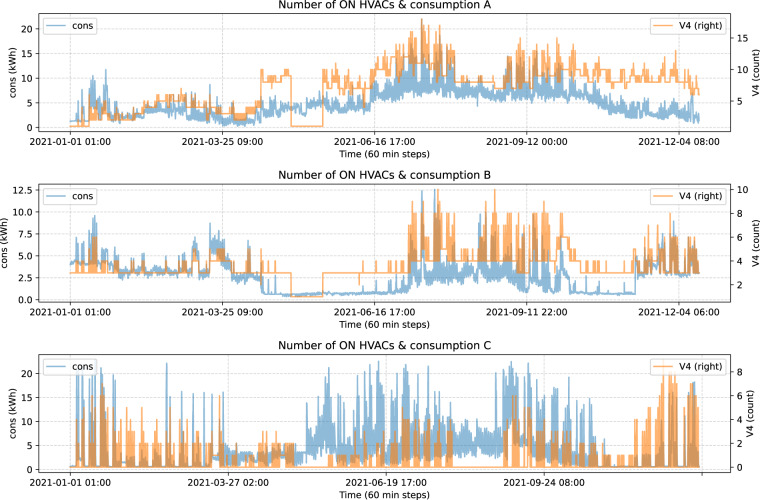
Number of on HVACs & consumption by block.

The merge in this section yields to a dataset used that most likely will be used to train and evaluate a consumption forecast model. The merge consists of consecutive merges with an inner join on the *Date* field. This merge allows us to plot the number of turned on HVACs along with consumption, as shown in Fig. 11.

Looking closely we can detect an strange behavior, for example around observation 3000 we see that the number of on HVACs is always 1, but consumption is far from constant. Lets see the correlation between those features in Table 2:

**Table 2:** Number of on HVACs & consumption correlation.

block	res_time	correlation
A	60T	0.677337
B	60T	0.553585
C	60T	0.266004

Trying to solve that we do a smoothing on V4 in blocks A and C, and in block A we remove observations where the number of HVACs is smaller or equal to 1. While in B and C we do not do anything else. Selecting which observations to smooth and to remove or not was a result of trial and error and looking for the higher correlation without removing too many observations.

**Fig. 12:**
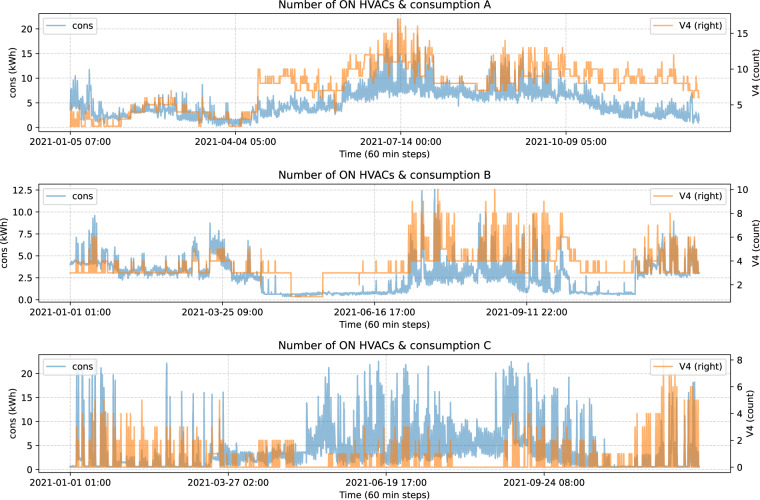
Number of on HVACs & consumption by block.

Now, in Fig. 12 the updated number of HVACs and consumption are represented:

And by doing that, correlation in blocks A and C is slightly improved, as we see en Table 3.

**Table 3:** Number of on HVACs & consumption correlation.

block	res_time	correlation
A	60T	0.713264
B	60T	0.553585
C	60T	0.0.313860

In this way, we have the optimal data to use for consumption predictions.

### Dataset merge 2. Temperature

This time, the merge yields a dataset that can be used to develop a temperature forecast model instead, with lower granularity than the previous one. To make temperature predictions that lower level of granularity is needed. This is a room level granularity and to achieve that we will use again the relationships between devices, HVACs and sensors. Also, in this case the resample time will be 10 minutes, not 60. Therefore, a step by step procedure will be done, divided in two parts.

*Note: files created in this section weight* ~ 250 *mb each*

The first part includes a code block with similar steps like the ones followed for consumption but going for the lower granularity, which involves grouping observations in a different way. From this we will get a file by block and resampling interval, in this case, we will get 3 (blocks A, B, C with 10 minutes resamples).

The second part merges those 3 files into one and orders samples by date, removes unnecessary columns and groups by room and block. Then, since temperature predictions are only useful in rooms with both temperature sensor and HVAC monitoring we look for the intersection of rooms which fulfill said requisites. Finally we create an index to homogenize all the observations and make sure that for every room in every block we have one observation every 10 minutes.

### CO_2_ and presence datasets

These two datasets are presented together because we think they could serve a similar purpose, which is knowing how the people inside the rooms behave, and this, coupled with HVAC operation or indoor temperature could provide even more additional information. One important aspect to keep into account is that the presence value is retrieved from a passive infrared motion sensor (PIR) which turns on when someone moves and then turns off after a certain time, usually between a few seconds and three minutes. Therefore, this data should not be considered as occupancy but as a reference to infer it later. One approach could be considering observations that have a 1 near zeros in a interval lower than 3 minutes as continuous presence. In this way, only the zeros that are far from ones would be considered as not presence and we would ignore the zeros that appear when the sensor is turned off but someone is actually in the room yet.

To prepare carbon dioxide data we merge it with its relationships to end up with data only from the rooms that we have more data. This step in specially important this time because there are other devices identifiers that do not belong to CO_2_ measurement devices, and therefore, they should not be taken into consideration. In addition to that, we perform a resample in 10 minute intervals grouping by mean value.

For the presence dataset the procedure is quite similar, with one particular aspect: when we do the resample we choose the max value instead of the mean, so if there was a 1, it takes the 1 regardless of having zeros in the given interval. Then, for the dates that it returns a *NaN* value we fill it with zeros because this happens when there is no register in that interval, which means that the sensor was not turned on and consequently the room is empty.

Finally, the two remaining datasets are merged with an inner join, having together the columns of: *block, room, date, V17 (CO*_2_*) and presence*.

Following all the previous steps, the data is prepared for a more in depth analysis by the reader if desired.

This descriptive analysis provides insight to get a deep understanding of the datasets presented. Knowing the data types of the features in each file allows us to perform proper operations, since working with datetime objects, integers or boolean values is quite different. The dataset size is also mentioned so the reader can consider if the code should run locally or otherwise, run on the cloud. Then, plotting of a feature versus time provides a simple representation of how a variable changes over time and brings the opportunity to visually get a first idea of why the parameters behave the way they do, and after that, more multivariable plots over time and correlation matrix are represented trying to explain how turning on and off the HVAC affects consumption and indoor temperature. Moreover, this process can be extrapolated and adapted to various types of datasets, especially those consisting of time series for a similar analysis before moving on to develop machine learning models or any other applications.

## Usage Notes

Some of the files that contain the data^25^ are quite large, therefore, the use of a specific tool to work with such files like *pandas* in Python or *data.table* library from in R is recommended.

## Data Availability

The code implementation was done in Python3 (3.8.3) using Jupyter notebook. The script that describes the step by step process followed is available at^25^.
